# The Dimeric Architecture of Checkpoint Kinases Mec1^ATR^ and Tel1^ATM^ Reveal a Common Structural Organization*

**DOI:** 10.1074/jbc.M115.708263

**Published:** 2016-04-28

**Authors:** Marta Sawicka, Paulina H. Wanrooij, Vidya C. Darbari, Elias Tannous, Sarem Hailemariam, Daniel Bose, Alena V. Makarova, Peter M. Burgers, Xiaodong Zhang

**Affiliations:** From the ‡ Section of Structural Biology, Department of Medicine, Imperial College London, South Kensington, London SW7 2AZ, United Kingdom and; the § Department of Biochemistry and Molecular Biophysics, Washington University School of Medicine, St. Louis, Missouri 63110

**Keywords:** checkpoint control, DNA damage response, nucleic acid enzymology, phosphatidylinositol kinase (PI Kinase), protein structure, serine/threonine protein kinase

## Abstract

The phosphatidylinositol 3-kinase-related protein kinases are key regulators controlling a wide range of cellular events. The yeast Tel1 and Mec1·Ddc2 complex (ATM and ATR-ATRIP in humans) play pivotal roles in DNA replication, DNA damage signaling, and repair. Here, we present the first structural insight for dimers of Mec1·Ddc2 and Tel1 using single-particle electron microscopy. Both kinases reveal a head to head dimer with one major dimeric interface through the N-terminal HEAT (named after Huntingtin, elongation factor 3, protein phosphatase 2A, and yeast kinase TOR1) repeat. Their dimeric interface is significantly distinct from the interface of mTOR complex 1 dimer, which oligomerizes through two spatially separate interfaces. We also observe different structural organizations of kinase domains of Mec1 and Tel1. The kinase domains in the Mec1·Ddc2 dimer are located in close proximity to each other. However, in the Tel1 dimer they are fully separated, providing potential access of substrates to this kinase, even in its dimeric form.

## Introduction

Mec1 (yeast orthologue of mammalian ATR (ataxia telangiectasia- and Rad3-related)) and Tel1 (yeast orthologue of ATM (ataxia telangiectasia-mutated)) belong to the phosphatidylinositol 3-kinase-related protein kinase (PIKK)^7^ family, the members of which control a plethora of cellular events including DNA damage response, DNA replication, growth control, and mRNA surveillance. Other members of the PIKK family include mammalian DNA-PKcs, mTOR (Tor1 and Tor2 in yeast), TRRAP, and SMG-1 (1, 2). PIKKs have a conserved domain architecture consisting of the FAT domain (named after the members, FRAP, ATM, and TRRAP), kinase domain, and FATC (FRAP, ATM, and TRRAP C terminus) domain at the C terminus, as well as HEAT repeats of variable lengths at the N terminus.

Because of the large size of these proteins, ranging from 2,368 amino acids for Mec1 to 4,128 amino acids for DNA-PKcs, these proteins are difficult to be purified in large quantities, thus hindering biochemical and structural analysis. There are a number of low resolution electron microscopy reconstructions available. These include a monomeric ATM with and without DNA bound at 30 Å resolution (3), dimeric ATM at 26 Å (4), DNA-PKcs with and without DNA bound, as well as DNA-bound DNA-PKcs·Ku70·Ku80 complex, all at ∼30 Å (5, 6), DNA-PKcs at 13 and 7 Å (7, 8), SMG-1 in complex with SMG-9 at 24 Å (9), SMG-1·UPF complexes (SMG-1·SMG-8·SMG-9 complex with UPF1 or UPF2) at a range of 17–22 Å (10, 11), mTOR complex 1 structure at 26 Å (12), and TOR complex 2 structure at 26 Å (13). The crystal structure of DNA-PKcs at 6.6 Å (14) provided the first medium resolution structural information on a full-length PIKK. Although not all regions are resolved, the structure reveals the large array of HEAT repeats arranged in a circular fashion, enclosing a large channel. Very recently, the crystal structure of the C-terminal domain of mTOR, containing the conserved FAT, kinase, and FATC regions was determined at 3.2 Å followed by the cryo-electron microscopy structure of the human mTOR complex 1 (mTORC1) at 5.9 Å revealing the dimeric organization of these domains (15, 16). Although the PIKKs feature a conserved amino acid sequence of the common domains, the known structures of monomers and dimers do not explain the precise domain organizations in a three-dimensional space for the various PIKKs. Furthermore, certain PIKKs such as ATM are believed to be active as monomers (17), whereas the mTOR structures reveal active dimers (13, 16).

In budding yeasts, Mec1 and Tel1 are apical enzymes in DNA damage checkpoint pathways. They detect DNA damage and temporarily halt cell cycle progression while elevating and activating DNA repair proteins (18–21). Mec1 and Tel1 phosphorylate numerous target proteins involved in cell cycle, DNA repair, and replication including Rad53, Chk1, Ddc1-Rad17-Mec3 (9-1-1 checkpoint clamp), RPA (replication protein A), and Rad51 (22–26). Furthermore, Mec1 and Tel1 also play important roles in telomere maintenance (27, 28). Current models suggest that Tel1 is recruited to double-stranded DNA ends, whereas Mec1 is recruited to RPA-coated single-stranded DNA, generated either by end resection during the DNA double-strand break repair or during replication fork reversal (25, 29). Mec1 and Tel1 show all the domains characteristic of PIKKs, including a PIKK regulatory domain at the C terminus, but lack a distinct FKBP12-rapamycin binding domain acting as a recruitment module (30). The PIKK regulatory domain is sandwiched between the kinase and FATC domains and is an essential regulatory element in ATR and ATM (31). The FATC domain is very well conserved and absolutely essential for kinase activity (31). It has been proposed to be the site for protein interactions and localization to damage sites (31). Tel1 contains an additional conserved N-terminal motif called TAN (Tel1/ATM N-terminal motif) that is important for telomere length maintenance and checkpoint signaling (32).

Similar to ATR, which forms a constitutive complex with its partner ATRIP, Mec1 also forms an integral complex with Ddc2 (also called Lcd1 or Pie1) (33–35). This interaction is coordinated by the C terminus of Ddc2 and the N terminus of Mec1 (35, 36). The N-terminal domain of Ddc2 has a coiled-coil domain important for oligomerization (37, 38) followed by a region involved in interactions with checkpoint activators like Dpb11 (mammalian TopBP1) (31, 39). The N-terminal domain of Ddc2 also features an acidic stretch that recognizes RPA, thereby recruiting ATR-ATRIP^Mec1·Ddc2^ complexes to single-stranded DNA (40). Remarkably, ATRIP^Ddc2^ interaction partners like TopBP1^Dpb11^ and RPA also interact with the kinase domain of ATR^Mec1^ via contacts with the PIKK regulatory domain or FATC regions (31, 41). A structure of Mec1·Ddc2 is thus required to understand how Mec1 and Ddc2 can coordinate various interactions important for Mec1 function.

The activation of Mec1·Ddc2^ATR-ATRIP^ complex and Tel1^ATM^is tightly regulated. Post-translational modifications such as acetylation in ATM (42) and autophosphorylation in ATM and ATR (17, 43) are shown to play important roles in activation. Activator proteins have been identified to substantially enhance mammalian PIKKs kinase activities such as TopBP1 for ATR-ATRIP and the Mre11·Rad50·Nbs1 complex for ATM (31, 44). Yeast Mec1·Ddc2 has a number of cell cycle-dependent activators, with the 9-1-1 clamp activating Mec1·Ddc2 in the G_1_ phase, Dpb11 together with the 9-1-1 clamp in the G_2_ phase, whereas Dna2, Dpb11, and the 9-1-1 clamp act in the S phase (45–48). Conserved hydrophobic residues in unstructured tails of the activator proteins are involved in bringing about the activation in Mec1·Ddc2 (49).

The precise activation mechanism is still unclear for both ATR^Mec1^ and ATM^Tel1^, especially regarding whether oligomerization plays a role in activation. For ATR-ATRIP, oligomerization of the ATRIP subunits is essential for activation (37, 38). However, ATM is proposed to undergo a dimer to monomer transition as a part of the activation mechanism. ATM also exists as active dimers on activation during oxidative stress; however, the dimers are formed by a disulfide bridge between the C-terminal FATC domains (50). Activation of both ATR and ATM is initiated by autophosphorylation, which has been proposed to happen in *trans* and hence might require the close association of both kinase domains (17, 43).

In this work, we provide the first structural information for dimers of Mec1·Ddc2 and Tel1 obtained using electron microscopy. These structures reveal the conformation of Mec1·Ddc2 and Tel1 in their preactivated states. Both Mec1·Ddc2 and Tel1 structures show a head to head dimer coordinated by the N-terminal HEAT repeats. Individual monomers of both Mec1 and Tel1 show a characteristic arm region formed by the N-terminal HEAT repeats and a head region formed by the FAT-kinase-FATC domains. A comparison of the Mec1·Ddc2 dimer with the Tel1 dimer shows a large difference in the distance between the head domains within the dimer, which are fully separated in Tel1 but in close proximity in the Mec1·Ddc2 complex.

## Experimental Procedures

### 

#### 

##### Protein Expression and Purification

The Mec1·Ddc2 complex was expressed and purified as previously described (51) with some modifications. The Mec1·Ddc2 complex tagged with an IgG binding domain (ZZ) was overexpressed in yeast from pBL904, and cells were harvested and lysed using buffer HEP^300^ (50 mm HEPES-KOH, pH 7.8, 300 mm KCl, 10% glycerol, 1 mm EDTA, 0.1% Tween 20, 0.02% C_12_E_10,_ 3 mm DTT, 5 mm reduced glutathione, 10 mm NaHSO_3_, 10 μm pepstatin A, 2 mm benzamidine, 10 μm leupeptin, 1 mm PMSF, 5 mm NaPP_i_, 10 mm β-glycerophosphate, 1 mm α-naphtylic acid, 5 mm NaF; superscript designates 300 mm NaCl). The cell lysate was adjusted to a pH of 7.4 and a conductivity corresponding to that of 200 mm KCl buffer and was clarified by ultracentrifugation at 35,000 rpm for 1 h in a 45 Ti rotor (Beckman Coulter). The supernatant was incubated with IgG beads (IgG-Sepharose 6 Fast Flow; GE Healthcare) for 3 h and subjected to four consecutive washes with buffer HEP^250^, HEP^300^, HEP^300^ supplemented with 10 mm magnesium acetate and 1 mm ATP, and HEP^400^. Mec1·Ddc2 was cleaved with HRV 3C protease and eluted.

The GST-tagged Tel1 was overexpressed in yeast from pBL602 and purified as previously described (51) with some modifications. Cells were harvested and lysed in buffer HEP^300^ (60 mm HEPES-KOH, pH 7.8, 40 mm potassium phosphate, pH 7.8, 10% glycerol, 300 mm KCl, 150 mm ammonium sulfate, 2 mm DTT, 0.1% Tween 20, 0.01% Nonidet P-40, 1 mm EDTA, 0.5 mm EGTA, 10 mm β-glycerophosphate, 1 mm α-naphtylic acid, 5 μm pepstatin A, 5 μm leupeptin, 3 mm NaHSO_3_, and 2 mm benzamidine). Ammonium sulfate precipitated protein was resuspended in buffer HEP^0^ and incubated with glutathione *S*-Sepharose beads for 3 h and subjected to four consecutive washes with buffer HEP^100^, HEP^100^ lacking the protease and phosphatase inhibitors hereinafter, HEP^100^ supplemented with 10 mm magnesium acetate and 1 mm ATP, and HEP^100^. The GST-tagged Tel1 was cleaved by HRV 3C protease and further purified over a heparin column and eluted in HEP^700^.

##### Gel Filtration Analysis

Mec1·Ddc2 was buffer-exchanged to 50 mm HEPES-KOH, pH 7.4, 200 mm KCl, 10% glycerol, 0.1% Tween 20, 0.01% Nonidet P-40, 1 mm EDTA, 1 mm EGTA, and 2 mm DTT using Amicon Ultra 0.5 columns (Merck Millipore) and run over a Superose 6 HR 10/30 gel filtration column (Pharmacia Biotech) at 0.3 ml/min. Tel1 was buffer-exchanged to 50 mm HEPES-KOH (pH 7.4), 200 mm KCl, 10% glycerol, 0.1% Tween 20, 0.02% C_12_E_10_, 1 mm EDTA, and 3 mm DTT and run over a Superose 6 Increase 5/150 GL gel filtration column (GE Healthcare) at 0.1 ml/min. Elution was monitored at 280 nm, and peak fractions were separated on an 8% SDS-PAGE gel to verify protein identity. Migration of the Mec1·Ddc2 complex and Tel1 was compared with that of the gel filtration standards (catalog no. 151-1901; Bio-Rad) to estimate the size of the complex.

##### Mec1 Kinase Activity Assays

10 nm Mec1·Ddc2 was incubated with 100 nm GST-Rad53-kd in 10-μl reactions containing 25 mm HEPES (pH 7.6), 100 mm NaCl, 8 mm magnesium acetate, 100 μg/ml BSA, 1 mm DTT, 100 μm ATP, 0.5 μCi of [γ-^32^P]ATP and 50 nm Dpb11, where indicated. Reactions were allowed to proceed for 10 min at 30 °C and stopped by addition of 4 μl of 5× SDS-PAGE loading dye. Samples were boiled for 5 min, separated on 8% SDS-PAGE gels, dried, and exposed to a phosphor screen (GE Healthcare).

##### Antibody Labeling of Mec1·Ddc2 and Tel1

16 nm Mec1·Ddc2 complex was labeled with 32 nm goat anti-Mec1 FATC, yS-20, polyclonal antibody (Santa Cruz Biotechnology) in 10-μl reactions containing 30 mm HEPES-KOH (pH 7.4), 150 mm NaCl, 2.5% glycerol, 0.25 mm EDTA, and 0.5 mm DTT. 20 nm Tel1 was labeled with the same anti-FATC antibody at 40 nm in 25-μl reactions containing 50 mm HEPES-KOH (pH 7.4), 220 mm NaCl, 2% glycerol, 1 mm EDTA, 0.5 mm EGTA, 1 mm DTT, 1 mm MgCl_2_, 0.02% Tween 20, 0.002% Nonidet P-40. The anti-Mec1, yS-20, antibody has been raised against the highly conserved C-terminal region of Mec1 (84% similarity with the Tel1 C-terminal region). The reactions were incubated on ice for 30 min before putting the labeled samples onto EM grids.

##### Negative Stain Electron Microscopy Data Collection

2 μl of 70 nm Mec1·Ddc2 (30 mm HEPES, pH 7.4, 200 mm NaCl, 5% glycerol, 0.5 mm EDTA, 1 mm DTT), 2 μl of 20 nm Tel1 (50 mm HEPES, pH 7.4, 150 mm NaCl, 5% glycerol, 1 mm EDTA, 1 mm DTT), and antibody-labeled samples at concentrations described above were deposited for 1 min on glow-discharged continuous carbon grids (TAAB Laboratory Equipment). The excess of liquid was blotted, and a 2-μl drop of 2% (v/v) uranyl acetate was added to the grids for 2 min. The excess liquid was blotted and left to air dry. Mec1·Ddc2, Tel1, and the anti-Mec1-labeled complexes of Mec1·Ddc2 were imaged in a Philips CM200 electron microscope operating at 200 kV and equipped with a TVIPS slow scan 4k × 4k CCD camera. Micrographs were manually collected at a nominal magnification of 50,000 with a pixel size of 1.76 Å/pixel corresponding to an electron dose of 20 e/Å^2^/s. All images were taken at a range of defocus from −2.5 to −3.5 μm. The antibody-labeled Tel1 sample was imaged in an FEI Tecnai F20 electron microscope operating at 200 kV and equipped with an FEI Falcon II CMOS direct electron detection camera. Micrographs were manually collected at a nominal magnification of 62,000 corresponding to a pixel size of 1.65 Å/pixel and an electron dose of 32 e/Å^2^/s.

##### Single Particle Negative Stain Image Processing

10,586 Mec1·Ddc2 and 7,350 Tel1 particles were picked using an e2boxer.py program of EMAN2 (52, 53). Defocus and astigmatism parameters were estimated using CTFFIND3 (54). Mec1·Ddc2 and Tel1 data sets were used for *ab initio* reconstructions using a standard multivariate statistical analysis/multireference alignment routine in IMAGIC-V (55). Briefly, all particles were band pass-filtered with a 200 Å high pass cutoff and a 10 Å low pass cutoff and subjected to reference-free alignment. Class averages were generated using multivariate statistical analysis allowing selecting distinctive classes, which were used as an initial reference set for multireference alignment. Euler angles were manually assigned to three class averages along distinctive views. The assigned angles served as a set of angular references to determine Euler angles for all class averages and subsequently create an initial three-dimensional model. Reprojections generated from the new model were used as a reference set to align particles and assign their orientation in three dimensions. Once the overall features of the Mec1·Ddc2 and Tel1 map were stabilized, 2-fold symmetry (C2) was applied onwards. Further refinement for Mec1·Ddc2 was carried out in RELION-1.3 (56). Particles were subjected to reference-free two-dimensional classification and subsequently reduced to 7,235 particles after removing poor quality particles. Three-dimensional reconstruction was generated by refining the Mec1·Ddc2 dimer model obtained using IMAGIC-V. The final reconstruction was obtained from 5,633 Mec1·Ddc2 particles at a resolution of 22.5 Å using the gold standard FSC (0.143 criterion) (57). The Tel1 reconstruction was further refined using reprojections from the 2-fold symmetrized model generated with IMAGIC-V and performing particle alignments and projection matching in SPIDER (58). Aligned particles were subjected to the multivariate statistical analysis routine to generate class averages in IMAGIC-V. Angular assignments of the class averages generated from IMAGIC-V, and back projection for refining the structure was performed in SPIDER iteratively. The final reconstruction was obtained from the full data set at a resolution of 21 Å (FSC = 0.5 criterion). The model was further verified using RELION 1.3 with the final reconstruction filtered to 40 Å that converged to a 24 Å similar model in RELION 1.3 using the gold standard FSC (0.143 criterion).

Because of high heterogeneity of the antibody-labeled Mec1·Ddc2 and Tel1, individual particles with a clearly visible extra density were selected manually. 1,951 antibody-labeled Mec1·Ddc2 particles were band pass-filtered with 220 and 20 Å cutoffs and subjected to a reference-free alignment in IMAGIC-V. Centered particles were subjected to the MSA routine followed by two-dimensional classification. Two-dimensional class averages that show a clear extra density were selected and compared with reprojections generated from the unlabeled model. 844 antibody-labeled Tel1 particles were also band pass-filtered with 220 and 20 Å cutoffs. Visual inspection between the two-dimensional class averages of anti-FATC Mec1·Ddc2/individual images of anti-FATC Tel1 particles and reprojections along the same Euler angles identified a number of antibody-labeled particles that show an extra density corresponding to the anti-FATC antibody. Positions of the bound antibodies were identified using triangulation methods.

##### Electron Cryomicroscopy Data Collection

Tel1 was diluted in a buffer optimized for cryofreezing while preserving the protein stability and integrity. The final buffer included 50 mm HEPES-KOH (pH 7.4), 210 mm KCl, 1% glycerol, 0.02% Tween 20, 0.002% Nonidet P-40, 1 mm EDTA, 0.5 mm EGTA, 1 mm DTT, and 0.5 mm MgCl_2_. Aliquots of 2 μl of Tel1 at a concentration of 20 nm were applied to glow-discharged holey carbon grids (Quantifoil Cu R2/2, 300 mesh) coated with an additional thin layer of carbon. After 30 s of incubation in a humidity chamber at 4 °C and 100% relative humidity, the grids were blotted for 6 s and flash frozen in liquid ethane using an FEI Vitrobot Mark III. The grids were imaged in an FEI Tecnai F20 electron microscope operating at 200 kV and equipped with an FEI Falcon II CMOS direct electron detection camera. Micrographs were manually collected at a nominal magnification of 62,000 corresponding to a pixel size of 1.65 Å/pixel and an electron dose of 32 e/Å^2^/s. Images were recorded at a defocus ranging from −1 to −4 μm.

##### Electron Cryomicroscopy Image Processing

All data were processed in RELION-1.4 (56). All the micrographs were corrected for CTF using CTFFIND3 (54). An initial data set of 20,519 particles, selected using both manual and autopicking routine in RELION, was extracted after binning the particles twice giving the pixel size of 3.3 Å/pixel. References for autopicking were generated using the initial ∼1,200 manually picked particles and performing a two-dimensional classification routine. The data set was further improved by multiple rounds of two-dimensional classification to remove poor quality particles, resulting in a reduced data set of 17,226 particles. Three-dimensional classification was then employed to extract structurally homogenous data for three-dimensional structure refinement. The negative stain-EM structure filtered to 60 Å was used as a starting model. The final data set included 8,962 particles for refinement of the Tel1 structure. Iterative rounds of three-dimensional classification protocol were used for refinement as described in the RELION tutorials for extremely difficult cases. Careful monitoring was carried out to avoid possible overfitting, and all interpretations of the structure have been carried out keeping this in mind. The final resolution estimated by this refinement protocol according to resolution estimates used in Bayesian approach (59) was 14.1 Å. A final round of refinement was then carried out for local refinement in Refine3D routine of RELION. The final reconstruction converged to a resolution of 19.2 Å using the gold standard FSC (0.143 criterion).

##### Fitting of mTOR Crystal Structures and Map Segmentation

A 3.2 Å crystal structure of the C-terminal mTOR (Protein Data Bank code 4JSV) without the FKBP12-rapamycin binding domain (residues 2,021–2,118) was docked into the EM density corresponding to the kinase domain of Mec1 and Tel1. A 5.9 Å atomic structure of mTOR (Protein Data Bank code 5FLC) modeled into the cryo-EM map of mTORC1 (EMD-3213) was docked into the EM densities of the Mec1·Ddc2 complex and Tel1. Prior to fitting, the densities corresponding to Raptor and mLST8 subunits and the FKBP protein were removed. The Mec1·Ddc2 and Tel1 three-dimensional maps were then segmented in Chimera (60), and the domain boundary was based on the molecular fitting.

## Results

### 

#### 

##### Mec1·Ddc2 and Tel1 Exist as Higher Order Oligomers

**FIGURE 1.**
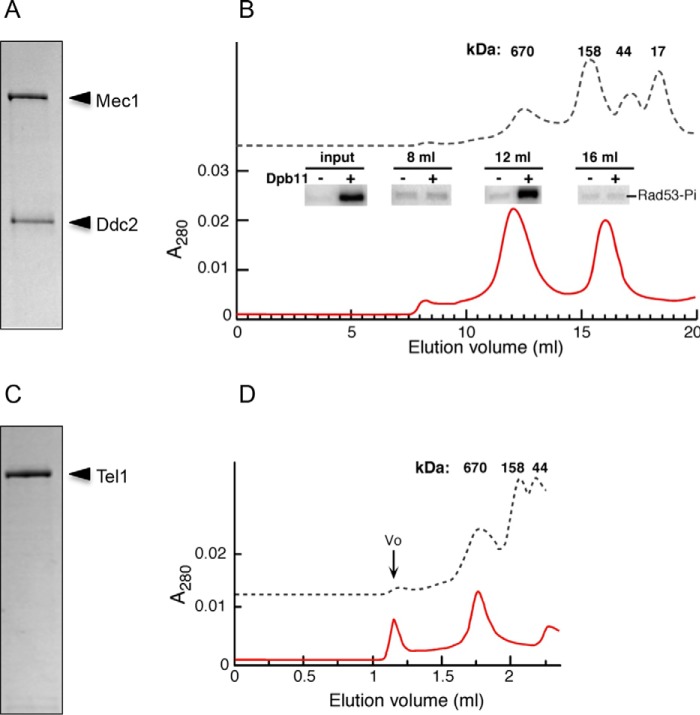
**Purification of Mec1·Ddc2 and Tel1.** *A*, SDS-PAGE of Mec1·Ddc2. *B*, gel filtration profile of Mec1·Ddc2 indicating that Mec1·Ddc2 exists as a dimer. The *inset* shows the kinase activity for specific fractions as indicated from the gel filtration profile. *C*, SDS-PAGE of Tel1. *D*, gel filtration profile of Tel1 indicating Tel1 exists as a dimer. *V*_0_ represents the aggregated material.

Mec1·Ddc2 was previously shown to form a stable complex of 360 kDa using analytical ultracentrifugation (51). However, these studies were carried out at high salt conditions (0.4 m KCl) to prevent aggregation. In this study, the protein was purified to high homogeneity with improved buffer conditions (Fig. 1*A*), and all the experiments have been carried out in physiological salt concentrations (100–200 mm KCl). We analyzed Mec1·Ddc2 on a Superose 6 HR 10/30 size exclusion column, and interestingly, we found that the majority of Mec1·Ddc2 elutes with a retention time corresponding to a complex of ∼700 kDa and is thus consistent with the molecular mass of the Mec1·Ddc2 dimer (720 kDa; Fig. 1*B*). Mec1 activity has been measured with *in vitro* kinase assays. The enhanced phosphorylation of Rad53 (with the active site mutated) and Rpa1 in the presence of Dpb11 was observed for the fraction containing the Mec1·Ddc2 dimer (Fig. 1*B*, *inset*). Therefore, we confirmed that Dpb11 could activate the oligomeric form of Mec1·Ddc2 used in our structural studies. Tel1 was purified to high homogeneity (Fig. 1*C*), analyzed on a Superose 6 Increase 5/150 GL size exclusion column, and eluted with a retention time corresponding to ∼700 kDa, which is consistent with the molecular mass of a Tel1 dimer (640 kDa; Fig. 1*D*).

##### Three-dimensional Reconstruction of Mec1·Ddc2 Dimer

**FIGURE 2.**
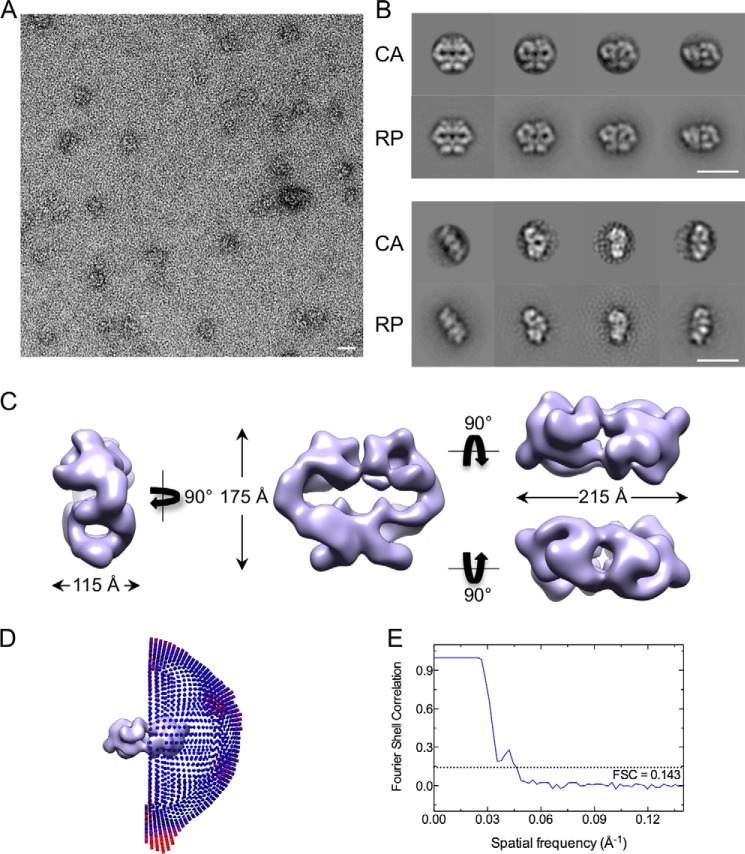
**Negative stain-EM three-dimensional reconstruction of Mec1·Ddc2 dimer.** *A*, a representative micrograph of negatively stained Mec1·Ddc2 particles. *B*, a comparison between two-dimensional CA and their corresponding RP generated from the negative stain three-dimensional model. *Scale bars* in *A* and *B* correspond to 200 Å. *C*, different views of the three-dimensional negative stain structure of Mec1·Ddc2 dimer. The *left panel* shows the side view, the *middle panel* shows the front view, and the *upper* and *lower right panels* show top and bottom views, respectively. *D*, Euler angle distribution of all particles used in refinement of the C2 symmetrized three-dimensional model. *E*, Fourier shell correlation curve used to calculate the model resolution of 22.5 Å according to the gold standard FSC (0.143 criterion).

To investigate the structural organization of Mec1·Ddc2, we used single particle negative stained EM to generate the three-dimensional model of the complex. Negative stained EM was selected because of relatively low sample concentration and poor contrast in cryo-EM. We observed two populations of particles that differed significantly in their sizes. The majority (81%) was consistent with the size of Mec1·Ddc2 dimer of 720 kDa (Fig. 2*A*). Based on the eigenimage analysis in IMAGIC-V (55), dimeric images displayed a 2-fold symmetry. Using angular reconstitution methodology implemented in IMAGIC-V, we obtained an *ab initio* three-dimensional reconstruction of Mec1·Ddc2 dimer and iteratively refined the model imposing 2-fold symmetry. The quality of model was assessed by a side by side comparison of the two-dimensional class averages (CA) with their corresponding reprojections (RP) generated from the three-dimensional model (Fig. 2*B*). The final negative stain Mec1·Ddc2 dimer model (Fig. 2*C*) was obtained from 5,633 particles. The distribution of Euler angles of all particles used in generating the C2 structure (Fig. 2*D*) covers all angular space. The three-dimensional refinement of the model converged at 22.5 Å (Fig. 2*E*). The dimeric Mec1·Ddc2 reconstruction (Fig. 2*C*) is 175 Å tall, 215 Å wide, and 115 Å thick, resembling a pretzel with tubular density encircling multiple cavities. We can clearly distinguish two regions, which have been referred to as the head and arm domain in ATM, DNA-PKcs, and SMG-1 reconstructions (3, 5, 9). Here, the head domain occupies the upper globular density, whereas the arm domain forms an elongated and curved shape and consists of tubular density regions encircling a cavity (Fig. 2*C*, *left panel*). The model reveals multiple interaction sites within the arm and the head domains. The arm of one monomer is intertwined with the arm of another monomer. Weak contacts are also observed between the two opposite head domains, suggesting their auxiliary roles in dimer formation and stabilization.

##### Three-dimensional Reconstruction of Tel1

**FIGURE 3.**
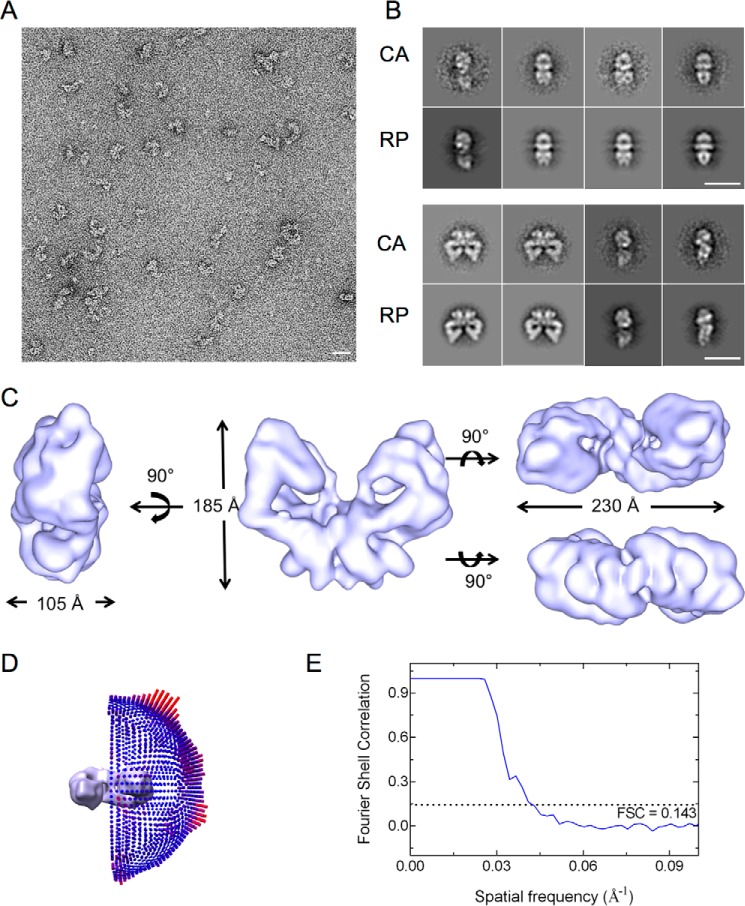
**Negative stain-EM three-dimensional reconstruction of Tel1 dimer.** *A*, a representative micrograph of negatively stained Tel1 particles. *B*, a comparison between two-dimensional CA and their corresponding RP generated from the negative stain three-dimensional model. *Scale bars* in *A* and *B* correspond to 200 Å. *C*, different views of the three-dimensional structure of Tel1 dimer. The *left panel* shows the side view, the *middle panel* shows the front view, and the *upper* and *lower right panels* show top and bottom views, respectively. *D*, Euler angle distribution of all particles used in refinement of the C2 symmetrized three-dimensional model. *E*, Fourier shell correlation curve used to calculate the model resolution.

Tel1 samples were initially analyzed using negative stained EM to build an initial model (Fig. 3). Single particles revealed a distinct 2-fold symmetry indicating that Tel1 existed mainly as dimers in the protein preparations. The initial model was generated using a data set of 7,350 particles using IMAGIC-V (55) and SPIDER (58). Reference-free alignments were carried during the initial stages of image processing, and subsequently angular reconstitution was utilized in building and refining the three-dimensional model. The quality of the model is assessed by the consistency between the CA and corresponding RP (Fig. 3*B*). The final negative stained three-dimensional model measures 185 Å tall, 230 Å wide, and 105 Å thick (Fig. 3*C*).

**FIGURE 4.**
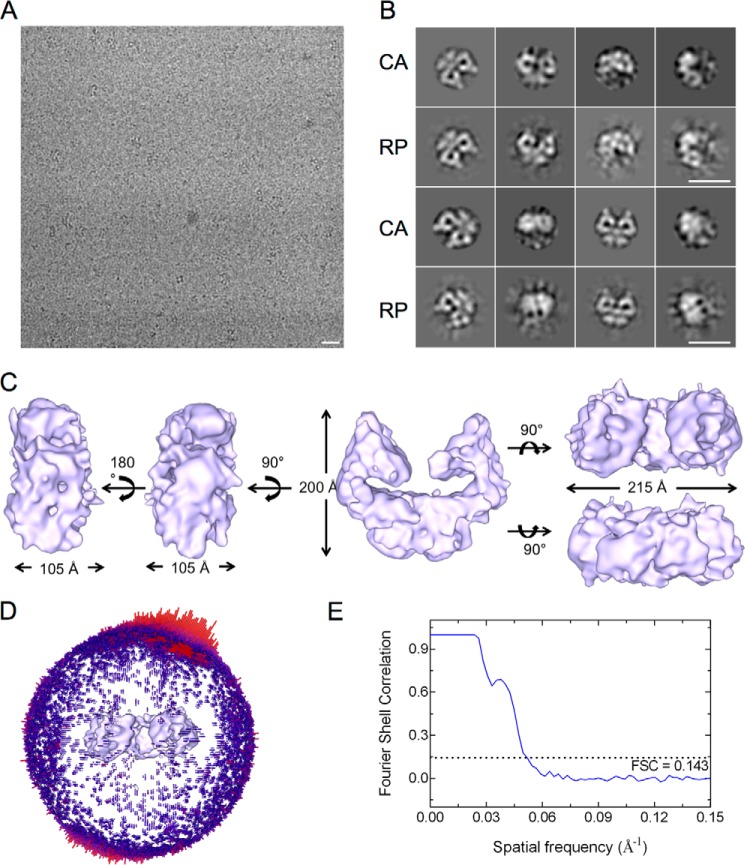
**Cryo-EM three-dimensional reconstruction of Tel1 dimer.** *A*, a representative micrograph of cryofrozen Tel1 particles on carbon-coated grids. *B*, a comparison between two-dimensional CA and their corresponding RP generated from the three-dimensional model. *Scale bars* in *A* and *B* correspond to 200 Å. *C*, different views of the three-dimensional structure of Tel1 dimer. The *two left panels* show the side views, the *middle panel* shows the front view, and the *upper* and *lower right panels* show top and bottom views, respectively. *D*, Euler angle distribution of all particles used in refinement of the non-symmetrized three-dimensional model. *E*, Fourier shell correlation curve used to calculate the model resolution of 19.2 Å according to the gold standard FSC (0.143 criterion).

Tel1 was subsequently vitrified in a buffer that was compatible for cryo-freezing and stability of the protein to perform cryo-EM analysis (Fig. 4*A*). An initial data set of 20,519 particles was selected by a combination of manual and autopicking in RELION (56). Multiple rounds of two-dimensional classification were used to remove poor quality particles, resulting in a reduced data set of 17,226 particles. Three-dimensional classification was used to extract structurally homogenous data for three-dimensional structure refinements. The negative stained EM model filtered to 60 Å was used as the starting model. Extraction of particles from the best model led to the final data set of 8,962 particles that was used to refine the Tel1 structure using the three-dimensional classification protocol. A further local refinement resulted in a final model at a resolution of 19.2 Å using the gold standard FSC (0.143 criterion). The quality of the three-dimensional reconstruction generated in RELION was assessed by comparing the class averages calculated in IMAGIC-V with the reprojections along the same angles, assigned in IMAGIC-V using references generated from the three-dimensional model (Fig. 4*B*).

The cryo-EM Tel1 structure (Fig. 4*C*) overall resembles the negative stain reconstruction (Fig. 3*C*). However, it is taller (200 Å *versus* 185 Å) and narrower (215 Å *versus* 230 Å) when compared with the negatively stained model. This could be due to the stain procedure, which could cause flattening artifact. The Tel1 structure starkly resembles the overall Mec1·Ddc2 dimer architecture. Each monomer displays the characteristic domains of the PIKKs with the arm and the head region. However, the two head domains in Tel1 are fully separated by 35 Å in the narrowest region and enclose a cavity ∼20 Å long and 125 Å wide. The Tel1 dimer structure displays no obvious steric hindrance for substrates to access the kinase domains.

##### Domain Assignment in Mec1·Ddc2 and Tel1

**FIGURE 5.**
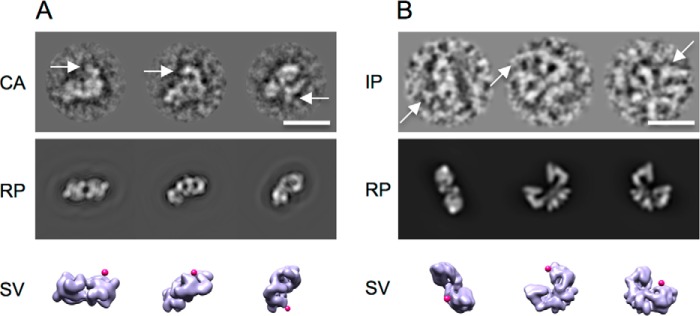
**Antibody labeling of the Mec1·Ddc2 and Tel1 reconstructions.** *A*, antibody labeling against the C terminus of Mec1. CA of anti-FATC antibody-labeled Mec1·Ddc2 show a clear extra density highlighted by *white arrows*. The density is missing in RP generated from the unlabeled Mec1·Ddc2 dimeric model along the same Euler angles as assigned to CA. *Magenta markers* indicate the location of the anti-FATC antibody as shown by surface views (*SV*) of Mec1·Ddc2 along the same Euler angles as in CA and RP. *B*, antibody labeling against the C terminus of Tel1. Individual particles (*IP*) show a clear extra density highlighted by *white arrows*. The density is missing in RP generated from the unlabeled Tel1 dimeric model along the same Euler angles as assigned to IP. *Magenta markers* indicate the location of the anti-FATC antibody as shown by surface views of Tel1 along corresponding angles. *Scale bars* correspond to 200 Å.

The three-dimensional reconstructions of both Mec1·Ddc2 and Tel1 display the head domain that is similar to head regions in other PIKK structures, where the FAT-kinase-FATC domains reside. We have used an antibody against the FATC domain to locate the FATC within Mec1·Ddc2 and Tel1 particle. Individual particles were carefully selected based on visual inspection. Only those representing the anti-FATC-labeled particle with additional density adjacent to it were selected. Subsequently we carried out the reference-free alignment and two-dimensional classification. Comparisons of the class averages with the reprojections generated from the Mec1·Ddc2 reconstruction located an extra density that corresponds to the anti-FATC antibody (Fig. 5*A*). For antibody-labeled Tel1 data, because of the preferential orientation and the low antibody labeling efficiency, there were insufficient data for reliable two-dimensional classification. However, because of the distinct front view, it is unambiguous that the anti-FATC antibody is located within the globular head domain (Fig. 5*B*). Thus the FATC domains in both Tel1 and Mec1·Ddc2 are located within the globular domain.

**FIGURE 6.**
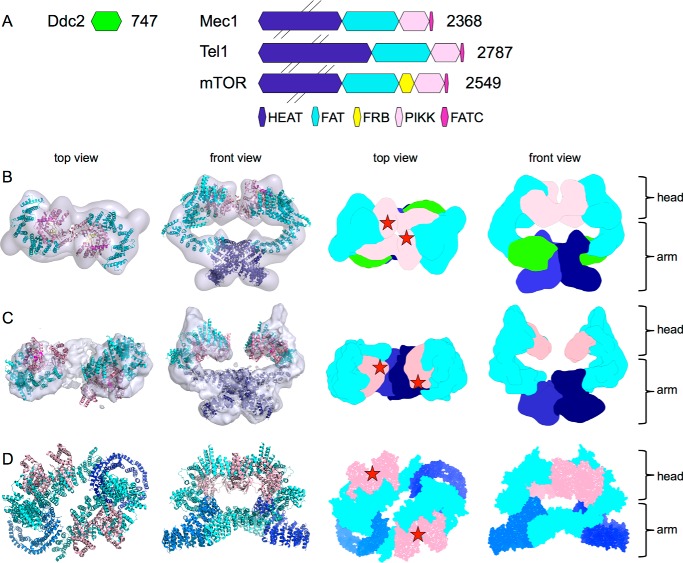
**Comparison of Mec1·Ddc2, Tel1, and mTORC1.** *A*, schematic domain arrangement of Ddc2, Mec1, Tel1, and mTOR. *B*, top and front views of the Mec1·Ddc2 EM reconstruction. *C*, top and front views of the Tel1 EM reconstruction with mTOR structures fitted in, as well as the top and front views of the segmented maps based on domain locations. *Pink*, FATC and kinase domain; *cyan*, FAT and HEAT repeats; *dark blue*, N-terminal HEAT repeats. The extra tubular density in Mec1·Ddc2 compared with Tel1, which accounts for Ddc2, is shown in *light green. D*, top and front views of the atomic model of mTOR (Protein Data Bank code 5FLC) fitted into the 5.9 Å cryo-EM structure of mTORC1 (EMD-3213). Densities corresponding to Raptor and mLST8 subunits of the complex, as well as the FKBP protein, have been removed. Top and front views of the segmented map of mTOR. *Red stars* indicate the positions of the active sites.

Fitting the crystal structure of the C-terminal mTOR (15) and the FAT-kinase-FATC domain of mTORC1 (16) into our EM models of Mec1·Ddc2 and Tel1 (Fig. 6, *B* and *C*) further supports that the cradle-shaped density of the head region consist of the FAT, kinase domain, and the FATC domain. Based on our molecular fitting, we could also suggest the possible location of the catalytic and activation loop within the cleft of the Mec1 and Tel1 kinase domain as shown for mTOR (Fig. 6*D*). The elongated tubular arm domains agree with the shape and the dimensions of the HEAT repeats, which dominate both the N-terminal domains of Tel1 and Mec1, as well as the majority of Ddc2. Upon fitting all domains of the full-length mTOR into the Mec1·Ddc2 model, we have observed an unassigned density extending from the N-terminal HEAT repeats of Mec1. This density also forms an elongated and tubular arm, indicating that it is a natural extension of the N-terminal Mec1 and therefore might correspond to the Ddc2 subunit. These tubular arm domains intertwine and form the dimer interface.

##### Comparison of Mec1·Ddc2 and Tel1

Mec1·Ddc2 and Tel1 structures display a similar dimer arrangement with the dimer interface formed by the N-terminal HEAT repeats of Tel1 and Mec1, as well as Ddc2 in Mec1·Ddc2 (Fig. 6). The overall dimensions of these two models are also comparable. However, the clear tubular density encircling a cavity in the arm region of the Mec1·Ddc2 reconstruction is missing in Tel1 (Figs. 2*C* and 3*C*). Monomeric Mec1·Ddc2 has a molecular mass of 360 kDa compared with 320 kDa of Tel1 (Fig. 6*A*); thus, it is likely that one of the tubular densities in the Mec1·Ddc2 reconstruction (Figs. 2*C*, *left panel*, and 6*B*, *front view*) accounts for Ddc2, as also concluded from our molecular fitting. Another striking difference between these two structures is the orientation of the head regions within the dimer. The two kinase domains are fully separated in Tel1, thereby suggesting no impediment for substrate recruitment in this model. On the other hand, the individual kinase domains in Mec1 are in close proximity, enclosing only a small cavity between the two domains.

## Discussion

Mec1 and Tel1 serve as key regulators of the DNA damage response. Together with other members of the PIKK family, they share a conserved domain arrangement of the N-terminal arrays of HEAT repeats followed by the C-terminal globular kinase flanked with the FAT and FATC domains.

Our EM reconstructions of Mec1·Ddc2 and Tel1 reveal their dimeric architecture and provide the first structural insights supporting earlier biochemical findings on Mec1·Ddc2^ATR-ATRIP^and Tel1^ATM^. Both models adopt a similar architecture with the head region comprising the FAT, kinase, and FATC domains, as well as the curved arm region consisting of large stretches of the N-terminal HEAT repeats. Our models are consistent with the 6.6 Å crystal structure of DNA-PKcs (14), as well as structures of other PIKKs such as SMG-1 (10). The DNA-PKcs structures show that the head region also consists of the kinase and FATC domain with additional HEAT repeats extending from the head domain and curving back onto it, encircling a large cavity. SMG1 is also shown to consist of a head region with a body comprising of tubular density that curves back toward the head domain. Although Mec1 exists in a complex with its obligatory binding protein Ddc2, it exhibits a similar structural organization. The C-terminal Ddc2 is predicted to contain HEAT repeats. Because Ddc2 interacts with the N-terminal Mec1, as previously reported biochemically (35), it might form a natural extension of the N-terminal HEAT repeats of Mec1. Based on our fitting of the 5.9 Å atomic model of mTOR (Fig. 6*B*) into the EM density of the Mec1·Ddc2 dimer, there is a remaining unassigned density extending from the N-terminal HEAT repeats of Mec1 that could accommodate the Ddc2 subunit. Then similar to DNA-PKcs and SMG-1, Ddc2 could possibly fold back into Mec1, positioning its N terminus in close proximity to the head region of Mec1. All PIKKs thus stabilize their C-terminal catalytic domains by using extended HEAT repeats, either within the same polypeptide chain as in Tel1^ATM^ and DNA-PKcs or by merging with an additional polypeptide chain such as Ddc2^ATRIP^.

Dimers of Mec1·Ddc2 and Tel1 represent a PIKK in a functional form that exhibits basal kinase activity prior to activation (Fig. 1*B*, *inset*). The oligomerization of ATR-ATRIP through the ATRIP coiled-coil region is required for localization into foci of radiation-induced damage sites, as well as stalled replication forks (37, 38). Ddc2 also contains a coiled-coil region and thereby likely contributes to the dimeric interface of Mec1·Ddc2. Our domain assignment of Mec1·Ddc2 (Fig. 6*B*) locates HEAT repeats at the dimeric interface. It is also seen in Tel1 structure that the HEAT repeats form the major dimer interface at the arm region (Fig. 6*C*). Similar findings have also been observed in the EM structures of mTORC1 (16) and TORC2 (13), and mutations within the HEAT repeats of Mec1 lead to defective DNA damage responses in the G_1_/S and intra-S checkpoints (61). Interestingly, we found that the dimeric interactions via HEAT repeats are likely to be stabilized by close vicinity of the FAT domain. Our Mec1·Ddc2 reconstruction shows that the kinase domains could also contribute to the dimer interface and could thus regulate substrate binding through the association or dissociation of the kinase domains without the requirement for a complete dissociation of the dimer. The recent cryo-EM reconstruction of mTORC1 (16) also shows a dimeric structure. The dimeric architecture is distinct from the one reported here. In Mec1·Ddc2 and Tel1, there is one major dimer interface, formed by HEAT repeats into a large intertwined density region. Although in mTORC1, at least two distinct dimer interfaces form by HEAT repeats, encircling a large cavity (Fig. 6*D*). In mTORC1, the two kinase domains are located similarly to that of Mec1 and Tel1, although the active sites are facing away from the dimer interface (Fig. 6, *B–D*). It is possible that, depending on the number of HEAT repeats, different forms of dimerization exist for the PIKKs. Alternatively, the different dimeric forms observed in our work and in mTORC1 could represent different functional states of the PIKKs.

A number of the PIKKs were reported to autophosphorylate via residues in the C-terminal domain as a molecular switch for their kinase activity (17, 43, 62, 63). Our reconstructions show that the two head domains can adapt very different conformations within the dimer, suggesting that one monomer can phosphorylate the adjacent monomer within the dimer. Upon DNA damage, ATM has been inferred to exist as a dimer in its inactive form, and dimer dissociation by autophosphorylation is associated with activation of kinase activity (17). However, in the presence of the oxidative stress, ATM dimerizes via a disulfide bridge in the FATC region and compensates for the loss of the Mre11·Rad50·Nbs1-dependent activity (50). The seemingly conflicting requirement for dimers is consistent with our results presented here. We show that kinase domains could contribute to the dimer interface but substrate binding and possibly activation do not necessarily require a complete dissociation of the dimer, which is formed primarily through the HEAT repeats. It is possible that phosphorylation might disrupt a subset of the dimer interface, thereby affecting the stability of the dimer, which could manifest in different oligomeric states *in vitro*.

Our data here suggest a putative model for Mec1 and Tel1. Although dimerization through these HEAT repeats brings the kinase domains of Mec1·Ddc2 in close proximity, perhaps suggesting that activation may require the separation of these domains, in Tel1, the kinase domains are separated by 35 Å. Therefore, activation of these PIKKs may proceed through a more complex mechanism rather than a simple separation of the kinase domains. There are certain mechanistic advantages to maintaining the dimer through the HEAT repeats anchor while allowing possible repositioning and activation of the kinase domains. A dimer could increase the interaction surface with cofactors or support cooperativity to allow a more efficient recruitment. Furthermore, a complete dissociation of dimers to monomers would imply that to deactivate Mec1 or Tel1 or to recycle it for other activities, reassociation into dimers would be required. A tethered dimer could ensure that it can be readily recycled between an inactive and an active state, allowing rapid multiple rounds of controlled activation/deactivation. However, understanding the exact activation mechanisms requires structural and biochemical data on a reconstituted complex involving the PIKK bound to its activator.

## Author Contributions

M. S., V. C. D., P. H. W., E. T., S. H., and D. B. designed and performed the experiments, and M. S., V. C. D., P. H. W., P. M. B., and X. Z. analyzed the data. P. M. B. and X. Z. conceived and designed the study. M. S. and V. C. D. performed the majority of electron microscopy, and M. S. did the antibody labeling studies, while D. B. collected the initial negative stain EM data for the Mec1·Ddc2 complex. P. H. W., E. T., A. V. M., and S. H. purified the proteins and performed biochemical analysis. M. S., V. C. D., X. Z., and P. M. B. wrote the manuscript.

## Footnotes

7 The abbreviations used are:

PIKK: phosphatidylinositol 3-kinase-related protein kinase
mTOR: mammalian target of rapamycin
CA: class average(s)
RP: reprojection(s)
FSC: Fourier shell correlation coefficient.
